# Association between the duration of mothers’ digital media use while with their children and two-year-old children’s development from the Japan Environment and Children’s Study

**DOI:** 10.1038/s41598-025-87430-9

**Published:** 2025-03-24

**Authors:** Yuka Ogata, Takatomo Matsumoto, Yuichi Suzuki, Toshie Nishigori, Aya Goto, Akiko Sato, Toma Fukuda, Karin Imaizumi, Hyo Kyozuka, Tsuyoshi Murata, Yuichi Nagasaka, Hidekazu Nishigori, Keiya Fujimori, Seiji Yasumura, Mitsuaki Hosoya, Koichi Hashimoto, The Japan Environment and Children’s Study Group, Koichi Hashimoto, Michihiro Kamijima, Shin Yamazaki, Maki Fukami, Reiko Kishi, Chiharu Ota, Chisato Mori, Shuichi Ito, Ryoji Shinohara, Hidekuni Inadera, Takeo Nakayama, Ryo Kawasaki, Yasuhiro Takeshima, Seiji Kageyama, Narufumi Suganuma, Shoichi Ohga, Takahiko Katoh

**Affiliations:** 1 Fukushima Regional Center for the Japan Environment and Children’s Study, 1 Hikarigaoka, Fukushima, 960-1295 Japan; 2 Department of Neuropsychiatry, Fukushima Medical University School of Medicine, 1 Hikarigaoka, Fukushima, 960-1295 Japan; 3 Department of Pediatrics, Fukushima Medical University School of Medicine, 1 Hikarigaoka, Fukushima, 960-1295 Japan; 4 Center for Integrated Science and Humanities, Fukushima Medical University, 1 Hikarigaoka, Fukushima, 960-1295 Japan; 5 Department of Obstetrics and Gynecology, Fukushima Medical University School of Medicine, 1 Hikarigaoka, Fukushima, 960-1295 Japan; 6 Department of Development and Environmental Medicine, Fukushima Medical Center for Children and Women, Fukushima Medical University Graduate School of Medicine, 1 Hikarigaoka, Fukushima, 960-1295 Japan; 7 Radiation Medical Science Center for the Fukushima Health Management Survey, Fukushima Medical University, 1 Hikarigaoka, Fukushima, 960-1295 Japan; 8 Department of Perinatology and Pediatrics for Regional Medical Support, Fukushima Medical University, 1 Hikarigaoka, Fukushima, 960-1295 Japan; 9 Nagoya City University, Nagoya, Japan; 10 National Institute for Environmental Studies, Tsukuba, Japan; 11 National Center for Child Health and Development, Tokyo, Japan; 12 Hokkaido University, Sapporo, Japan; 13 Tohoku University, Sendai, Japan; 14 Chiba University, Chiba, Japan; 15 Yokohama City University, Yokohama, Japan; 16 University of Yamanashi, Chuo, Japan; 17 University of Toyama, Toyama, Japan; 18 Kyoto University, Kyoto, Japan; 19 Osaka University, Suita, Japan; 20 Hyogo Medical University, Nishinomiya, Japan; 21 Tottori University, Yonago, Japan; 22 Kochi University, Nankoku, Japan; 23 Kyushu University, Fukuoka, Japan; 24 Kumamoto University, Kumamoto, Japan

**Keywords:** Digital media use, Maternal impact, Language development, Early development, Mother–child relationship, Neuroscience, Psychology

## Abstract

Few studies have evaluated the association between the duration of mothers’ digital media use (including mobile phones, tablets, and PCs) while with their children and children’s development. Using data from the Japan Environment and Children’s Study, we explored this relationship through multiple regression analysis. Self-administered questionnaires measured the duration of mothers’ digital media use. Developmental outcomes were assessed using the Kyoto Scale of Psychological Development 2001 (KSPD), administered to two-year-olds by trained examiners. The KSPD assessed three domains: postural-motor (fine and gross motor abilities), cognitive-adaptive (non-verbal cognitive capacity and visual-spatial comprehension), and language-social (interpersonal relationships, socialization, and verbal abilities). The analysis included 3,786 mother–child pairs with complete exposure data, outcomes, and covariates. The children of mothers who used digital media for one hour or more exhibited lower language-social development quotients compared with those whose mothers did not use digital media while with them. Furthermore, the children of mothers who used digital media for two hours or more showed a lower total developmental quotient compared with those whose mothers did not use digital media. The use of digital media by mothers for more than one hour per day while with their children is thus negatively associated with language development in two-year-olds, while use for more than two hours is negatively associated with children’s development.

## Introduction

Digital media, including on mobile phones, tablets, and PCs, is frequently utilized by many mothers. Average daily mobile phone use by mothers is reported to be 1.65 h in Germany, 3.17 h in Thailand, 2.67 h in South Korea, and 2.23 h in Japan^1–4^. Digital media offers several benefits for mothers of young children, such as access to child-rearing information, opportunities for interaction, and means to alleviate stress and boredom^3,5^. However, there are associations between digital media use and mental health issues such as depression, anxiety, stress, low self-esteem, loneliness within relationships, low emotional regulation and mindfulness, attachment challenges, and negative parenting^5–10^. Moreover, the use of digital media by mothers is linked to reduced verbal and non-verbal interactions with, and responsiveness to, their children^11–13^. Excessive smartphone dependence may result in mother–child attachment disorders, maternal neglect, and increased aggression toward children^3,8^. High media usage by mothers also correlates with emotional and conduct problems, as well as hyperactivity and inattention, in children^1^. Additionally, maternal smartphone dependency is associated with aggression, oppositional behavior, and emotional instability in children, thus leading to negative developmental tendencies such as impaired self-expression and self-regulation^3^.

Previous studies have reported no association between maternal digital media use time and children’s developmental outcomes^2,14^. In contrast, one study examined the association between the likelihood of parents using digital media during common everyday routines with their children and children’s language development^15^. Although this study found no association between parents’ digital media use and children’s language development, it did reveal a high likelihood that parents’ use of digital media during daily routines with their children is negatively correlated with children’s language development^15^. Previous research on the association between mothers’ digital media use time and children’s developmental outcomes has focused on the daily duration of mothers’ digital media use, rather than limiting focus to time spent with children^2,14,15^. Therefore, the results may be different if we examine the association between mothers’ digital media use and children’s developmental outcomes when we focus only on the mothers’ digital media use while with their children. Previous studies have shown that quality early mother–infant interactions, especially maternal responsiveness, are critical predictors of a child’s social, emotional, cognitive, and linguistic development^11,16–18^. Parental smartphone use reduces parental sensitivity and responsiveness, negatively impacting parent–child interactions^19^. The frequent interruption of interpersonal relationships and shared time by the use of technology devices—referred to as “technoference”^20,21^—has been suggested to hinder word learning and interaction outcomes^22^. Therefore, it is important to consider the association between mothers’ digital media use and children’s development by focusing on mothers’ time spent with their children, when their interactions could be interrupted.

In this study, in addition to the mother’s mental health (neuropsychiatric disorders, psychological distress, bonding failure), which is related to digital media use as mentioned above, we also included the child-rearing environment (e.g., frequency of reading to the child, number of hours per day the child is allowed to watch TV or DVDs) and socioeconomic status as adjustment variables. No previous studies have examined the association between digital media use and child development using multiple adjustment variables. Furthermore, no objective evaluations have been reported on the association between mothers’ use of digital media and their children’s developmental outcomes in Japan.

The Japan Environment and Children’s Study (JECS)—a large-scale birth cohort survey—has been conducted since 2011, targeting 100,000 pairs of mothers and children nationwide in Japan^23,24^. Trained inspectors utilize the Kyoto Scale of Psychological Development 2001 (KSPD) to assess the development of two-year-old children in a Sub-Cohort Study of 5% of JECS participants, selected by random sampling^25^.

The first 1,000 days from conception to age two are deemed crucial for establishing the physical, cognitive, and socioemotional foundations for subsequent life, as the period with the greatest brain plasticity^26^. During this time, the brain develops the most rapidly and can be greatly influenced by nutrition, protection (e.g., from toxic stress, violence, abuse, and neglect), and responsive stimulation (talk, play, reading, and responsive interaction with a loving adult)^27^. Additionally, the developmental status at age two can predict later school performance and socioeconomic status in adulthood^28^.Therefore, the age of two is an important milestone for assessing development and the nurturing environment of the mother and child.

The aim of this study was to investigate the association between the amount of time mothers spent using digital media while with their children and their children’s developmental outcomes at age two, as assessed by the KSPD, for a large cohort. The developmental outcomes used were motor, cognitive, and language development, as well as overall development, which included all three outcomes. In addition, mother’s mental health, child-rearing environment, and socioeconomic status were used as control variables.

## Materials and methods

### JECS

The JECS aims to elucidate the effects of environmental factors on children’s growth and development. The protocol for this study has been published in extant studies^23,24^. From January 2011 to March 2014, participants were recruited from 15 Regional Centers nationwide; 100,000 pairs of mothers and children were registered and will be followed up until the children turn 13.

A sub-cohort study was planned for 5% of the 100,000 JECS participants through random sampling to obtain more detailed information via an objective evaluation method (such as medical examinations by pediatricians and psychological development assessments by trained staff)^25^.

The JECS was conducted in accordance with the guidelines of the Declaration of Helsinki. The JECS protocol was reviewed and approved by the Ministry of the Environment’s Institutional Review Boards on Epidemiological Studies (No. 100910001) and the Ethics Committees of all participating institutions. Written informed consent was obtained from all of the participants or their legal guardians.

### Design and participants

**Fig. 1:**
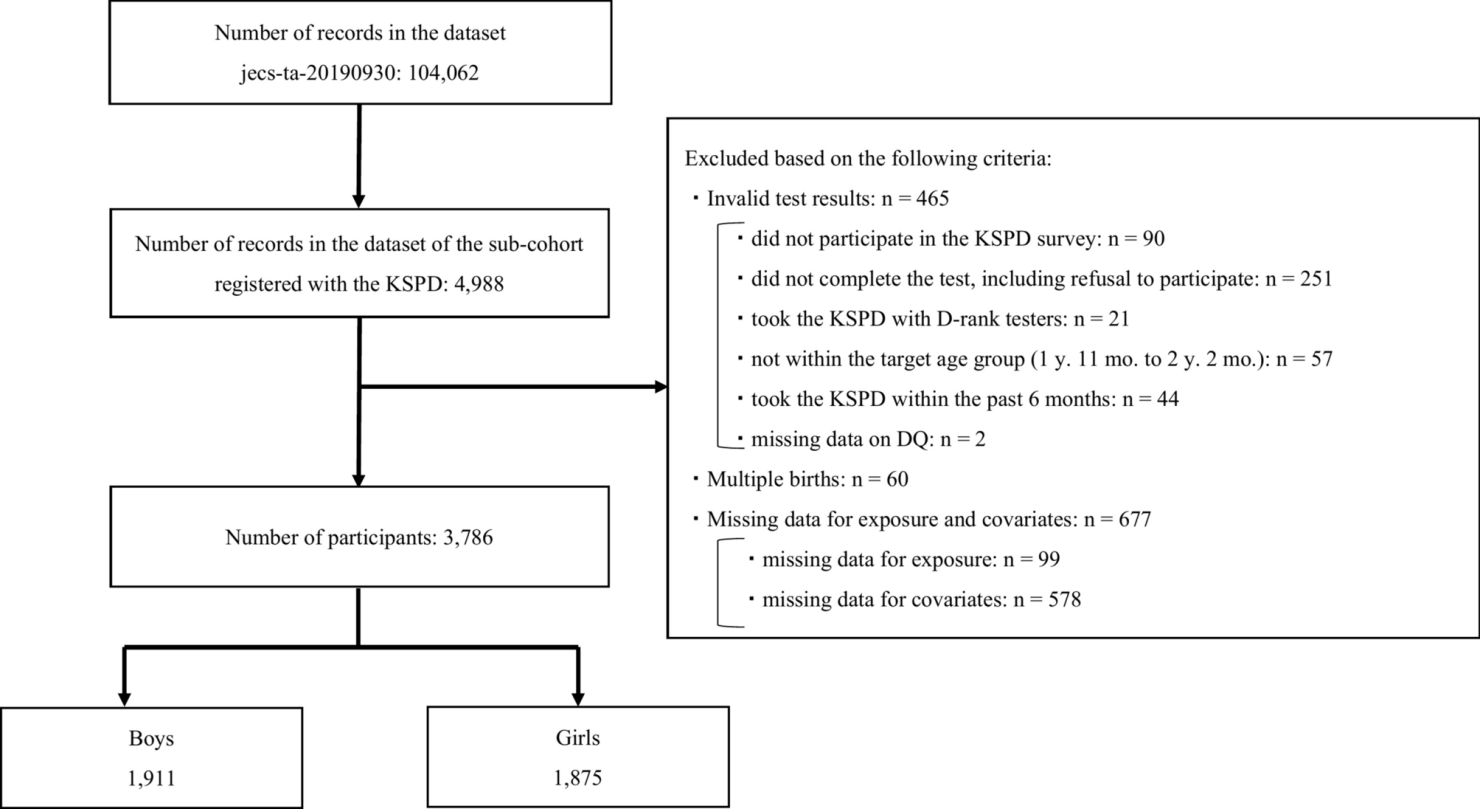
Flow chart for the selected participants. We used data from 104,062 pairs of pregnant women and fetuses, including multiple births. Of these, the Kyoto Scale of Psychological Development 2001 (KSPD) was administered to participants in the sub-cohort at age two years, yielding data on 4,988 pairs. After excluding invalid KSPD results, multiple births, and those with missing data related to exposure and covariates, we finally analyzed 3,786 pairs.

For our study, we adopted data on the participants included in the JECS Sub-Cohort Study. We used dataset jecs-ta-20190930, which was issued in October 2019 and revised in November 2022. The dataset contains 104,062 pairs of parent–child data points (including miscarriages and stillbirths) collected from gestation to age three. In a sub-cohort study, the KSPD was conducted at two years of age, providing data on 4,988 pairs. The exclusion criteria were invalid test results, multiple births, and missing exposure or covariate data (Fig. 1). As a result, we targeted 3,786 pairs of parents and children.

### Exposure: Mothers’ digital media use duration

To investigate the amount of time mothers spent using digital media while with their children, we used responses from a self-administered parental questionnaire mailed to caregivers when the child was one year and 11 months old. The questionnaire inquired about the number of hours per day the respondents spent on the computer, mobile phone, tablet, or video games while with their children. Respondents were asked to choose from “none”, “less than one hour”, “one hour or more but less than two hours”, “two hours or more but less than four hours”, and “four hours or more”. Using these caregiver responses and referring to previous studies^15^, the responses “none”, “less than one hour”, “one hour or more but less than two hours”, and “two hours or more” were classified into four groups. Unanswered exposure questions and non-mother responses were excluded due to being considered missing exposure data.

### Outcomes: Children’s motor, cognitive, language and overall development

Children’s developmental outcomes were assessed using the KSPD, which evaluates three domains: postural-motor (P-M), cognitive-adaptive (C-A), and language-social (L-S). P-M corresponds to fine and gross motor abilities; C-A to non-verbal cognitive capacity and visual-spatial comprehension; and L-S to interpersonal relationships, socialization, and verbal abilities^29^. The KSPD is an individual, standardized, and developmental face-to-face test that contains 328 items for Japanese children and adults^30^. In this study, the KSPD was conducted only with children. Based on the KSPD manual, the scores for these three areas and total scores were converted to developmental ages using a conversion table. In addition, developmental age was divided by the corrected chronological age (chronological age corrected based on gestational day 280, considering the influence of gestational age) and multiplied by 100 to calculate the developmental quotient (DQ)^30,31^. Thus, DQs (continuous variables) for P-M, C-A, L-S, and the total were calculated.

The KSPD has been compared to other face-to-face developmental tests, such as the Bayley Scales of Infant and Toddler Development 3rd Edition (Bayley-III) and Denver Developmental Screening Test II (DDST-II). The relationships between the corresponding pairs of KSPD domains and Bayley-III scores were analyzed and correlations were found: C-A DQ and cognitive composite scores, L-S DQ and language composite scores, and P-M DQ and motor composite scores^31^. The correlation between the KSPD DQ and the DDST-II has been analyzed, and correlations between the P-M DQ and gross motor, C-A DQ and fine motor-adaptive/personal-social, and L-S DQ and language have been confirmed^32^. The KSPD DQ had a significant positive correlation with IQ on the Tanaka-Binet Intelligence Scale (Japanese version of the Stanford-Binet Intelligence Scale); the total DQ had the highest correlation^29^.

As part of the JECS, the KSPD is used to assess two-year-olds (ranging from one month before to three months after they turn two). Certified inspectors conduct the KSPD following standardized procedures and quality control measures^33,34^.

### Statistical analysis and covariables

The characteristics of the participants were analyzed using descriptive statistics. Using the χ^2^ and t-tests, we examined the differences in mothers’ duration of digital media use and KSPD DQ by children’s sex. Finally, we examined the association between mothers’ digital media use and the development of their two-year-old children using multiple regression analysis, and conducted stratification analysis by children’s sex. In the multiple regression analysis, we adjusted for various covariates that could affect maternal digital media duration and child development: maternal age at delivery (continuous variable); marital status (married, common law marriage/divorced, widowed, other); maternal psychological distress (The 6-item Kessler Psychological Distress Scale [K6] ≧5: no/yes)^35^; neuropsychiatric disorders in mothers (no/yes); maternal bonding failure (The Japanese version of Mother-to-Infant Bonding Scale [MIBS-J] ≧5: no/yes)^36^; maternal education level (< 10; 10 to < 13; ≧13); paternal education level (< 10; 10 to < 13; ≧13); maternal occupation (no/yes); household income (× 10^3 yen/year; < 2,000; 2,000 to < 4,000; 4,000 to < 6,000; ≧6,000); family structure (nuclear/extended); partner’s frequency of participating in child care activities (never, seldom/sometimes/often, very often); frequency of meeting with other parents of same-aged children (seldom, 1–3 times a month, 1–2 times a week, 3–4 or more times a week); number of children including the participant (1, 2, ≧3); presence of physical anomalies (no/yes)^37^; attendance at a daycare center or nursery (no/yes); frequency of reading to the child (1–3 times a month or less, 1–2 times a week, 3–4 times a week, 5 or more times a week); number of hours per day the child is allowed to watch TV or DVDs (< 1, 1 to < 2, ≧2); and outdoor playtime during summer (May to September) and winter (November to February) (hours per day) (< 1, ≧1). Data on these covariates were gathered through responses to a self-report questionnaire and from medical record transcripts. Statistical significance was set at p < 0.05. All statistical analyses were conducted using SPSS 28.0 for Windows (SPSS Inc., Chicago, IL, USA).

## Results

Table 1 presents the characteristics of the 3,786 parent–child pairs included in the study. Regarding the daily duration of mothers’ digital media use while with their children, 15.4% reported “none”, while 63.2%, 16.5%, and 4.9% reported “less than one hour”, “one hour or more but less than two hours”, and “two hours or more”, respectively. The results of the χ^2^ test indicated no significant differences in the duration of mothers’ digital media use based on the sex of the child (χ^2^ (3) = 2.30, *ns*).

**Table 1:** The characteristics of the participants (N = 3,786).

		Overall		Boys		Girls		Reference for multiple regression analysis	
		(n = 3,786)		(n = 1,911)		(n = 1,875)			
		n	%	n	%	n	%		
Maternal age at delivery (years)	Mean (SD)	32	(4.8)	32	(4.7)	32	(4.9)	Continuous variable	
	Median (IOR)	32	(29–36)	32	(29–36)	32	(29–36)		
Marital status	Married, common law marriage	3,758	99.3	1,894	99.1	1,864	99.4	ref	
	Divorced, widowed, other	28	0.7	17	0.9	11	0.6		
Maternal psychological distress (K6 ≥ 5)	No	2,905	76.7	1,461	76.5	1,444	77.0	ref	
	Yes	881	23.3	450	23.5	431	23.0		
Neuropsychiatric disorders in mothers	No	3,416	90.2	1,732	90.6	1,684	89.8	ref	
	Yes	370	9.8	179	9.4	191	10.2		
Maternal bonding failure (MIBS-J ≥ 5)	No	3,362	88.8	1,668	87.3	1,694	90.3	ref	
	Yes	424	11.2	243	12.7	181	9.7		
Maternal education level (years)	< 10	119	3.1	54	2.8	65	3.5		
	10 to < 13	1,001	26.4	484	25.3	517	27.6	ref	
	> 13	2,666	70.4	1,373	71.8	1,293	69.0		
Paternal education level (years)	< 10	196	5.2	96	5.0	100	5.3		
	10 to < 13	1,240	32.8	613	32.1	627	33.4	ref	
	> 13	2,350	62.1	1,202	62.9	1,148	61.2		
Maternal occupation	No	2,016	53.2	1,005	52.6	1,011	53.9	ref	
	Yes	1,770	46.8	906	47.4	864	46.1		
Household income (× 1000 yen/year)	< 2,000	154	4.1	66	3.5	88	4.7		
	2,000 to < 4,000	1,215	32.1	610	31.9	605	32.3		
	4,000 to < 6,000	1,302	34.4	673	35.2	629	33.5	ref	
	> 6,000	1,115	29.5	562	29.4	553	29.5		
Family structure	Nuclear	3,080	81.4	1,545	80.8	1,535	81.9	ref	
	Extended	706	18.6	366	19.2	340	18.1		
Partner’s frequency of participating in childcare activities	Never, seldom	253	6.7	135	7.1	118	6.3		
	Sometimes	991	26.2	487	25.5	504	26.9		
	Often, very often	2,542	67.1	1,289	67.5	1,253	66.8	ref	
Frequency of meeting with other parents of same-aged children	Seldom	1,161	30.7	589	30.8	572	30.5	ref	
	1–3 times a month	1,341	35.4	704	36.8	637	34.0		
	1–2 times a week	776	20.5	390	20.4	386	20.6		
	3–4 or more times a week	508	13.4	228	11.9	280	14.9		
Number of children including the participant	1	1,522	40.2	783	41.0	739	39.4	ref	
	2	1,517	40.1	763	39.9	754	40.2		
	> 3	747	19.7	365	19.1	382	20.4		
Sex	Boys	1,911	50.5	1,911	100	0	0		
	Girls	1,875	49.5	0	0	1,875	100		
Gestational age (weeks)	< 37	131	3.5	75	3.9	56	3.0		
	> 37	3,655	96.5	1,836	96.1	1,819	97.0		
Presence of physical anomalies	No	3,452	91.2	1,723	90.2	1,729	92.2	ref	
	Yes	334	8.8	188	9.8	146	7.8		
Attendance at a daycare center or nursery	No	1,993	52.6	992	51.9	1,001	53.4	ref	
	Yes	1,793	47.4	919	48.1	874	46.6		
Frequency of reading to the child	1–3 times a month or less	542	14.3	291	15.2	251	13.4	ref	
	1–2 times a week	1,042	27.5	525	27.5	517	27.6		
	3–4 times a week	954	25.2	476	24.9	478	25.5		
	5 or more times a week	1,248	33.0	619	32.4	629	33.5		
Number of hours per day the child is allowed to watch TV or DVDs	< 1	1,039	27.4	539	28.2	500	26.7	ref	
	1 to < 2	1,590	42.0	807	42.2	783	41.8		
	> 2	1,157	30.6	565	29.6	592	31.6		
Outdoor playtime during summer and winter	< 1	2,609	68.9	1,311	68.6	1,298	69.2	ref	
	> 1	1,177	31.1	600	31.4	577	30.8		
*Exposure*									*P* value^a^
Mother’s digital media use duration (hours)	None	583	15.4	310	16.2	273	14.6	ref	.5
	< 1	2,391	63.2	1,198	62.7	1,193	63.6		
	1 to < 2	626	16.5	307	16.1	319	17.0		
	> 2	186	4.9	96	5.0	90	4.8		
*Outcome*									*P* value^b^
KSPD									
Total DQ	Means (SD)	94	(10.4)	92	(9.9)	96	(10.5)		< .001
	Median (IOR)	94	(88–101)	92	(86–98)	96	(90–103)		
P-M DQ	Means (SD)	94	(18.0)	93	(18.1)	94	(17.8)		.012
	Median (IOR)	85	(81–111)	84	(80–111)	91	(81–111)		
C-A DQ	Means (SD)	96	(12.6)	94	(12.0)	98	(12.8)		< .001
	Median (IOR)	95	(88–103)	93	(86–101)	97	(90–107)		
L-S DQ	Means (SD)	92	(14.9)	89	(14.7)	96	(14.5)		< .001
	Median (IOR)	93	(82–101)	89	(80–98)	95	(86–105)		

Kyoto Scale of Psychological Development (KSPD) 2001, Developmental Quotient (DQ), Postural-Motor (P-M), Cognitive-Adaptive (C-A), Language-Social (L-S), standard deviation (SD), interquartile range (IQR), The 6-item Kessler Psychological Distress Scale (K6; scores range from 0 to 24), The Japanese version of Mother-to-infant Bonding Scale (MIBS-J; scores range from 0 to 30).

^a^Sex differences were analyzed using Chi-square test.

^b^Sex differences were analyzed using Student’s t-test.

The average (SD) postural-motor (P-M), cognitive-adaptive (C-A), language-social DQs, and total DQ were 94 (18.0), 96 (12.6), 92 (14.9), and 94 (10.4), respectively. The results of the t-test showed significant differences in DQ and sex (P-M DQ (*t*(3784) = 2.51, *p* = 0.012), C-A DQ (*t*(3784) = 10.47, *p* < 0.001), L-S DQ (*t*(3784) = 12.93, *p* < 0.001), and total DQ (t(3784) = 12.55, *p* < 0.001)). P-M, C-A, L-S and total DQ were higher in girls than in boys. In this study, girls had a more advanced development than boys at the age of two years.

Table 2 presents the results of the multiple regression analysis of mothers’ digital media use duration and DQ after adjusting for covariates. No multicollinearity was detected in this analysis (variance inflation factor (VIF) < 3). Compared with the “none” group, the “one hour or more but less than two hours” group (B = −3.07; 95% CI −4.81 to −1.33) and the “two hours or more” group (B = −5.24; 95% CI −7.76 to −2.73) exhibited lower L-S DQs. Additionally, compared with the “none” group, the total DQ was lower for the “two hours or more” group (B = −2.07; 95% CI −3.86 to −0.28).

**Table 2:** Multiple regression analysis of mothers’ digital media use duration and KSPD scores (N = 3,786).

		Overall (n = 3,786)^a^						Boys (n = 1,911)^a^						Girls (n = 1,875)^a^					
		B	β	95% CI			*p*	B	β	95% CI			*p*	B	β	95% CI			*p*
Total DQ																			
Mothers’ digital media use duration (hours)	None	ref						ref						ref					
	< 1	−0.08	−.004	−1.02	–	0.87	.87	0.23	.01	−1.00	–	1.46	.71	−0.63	−.03	−2.04	–	0.77	.38
	1 to < 2	−0.87	−.03	−2.11	–	0.36	.17	−0.70	−.03	−2.34	–	0.93	.40	−1.46	−.05	−3.27	–	0.34	.11
	> 2	−2.07	−.04	−3.86	–	−0.28	.02	−1.67	−.04	−4.03	–	0.68	.16	−2.50	−.05	−5.11	–	0.12	.06
P-M DQ																			
Mothers’ digital media use duration (hours)	None	ref						ref						ref					
	< 1	−0.14	−.004	−1.79	–	1.52	.87	0.91	.02	−1.38	–	3.21	.44	−1.46	−.04	−3.86	–	0.94	.23
	1 to < 2	−0.49	−.01	−2.65	–	1.67	.66	1.37	.03	−1.67	–	4.42	.38	−2.75	−.06	−5.83	–	0.34	.08
	> 2	−2.34	−.03	−5.47	–	0.79	.14	−1.18	−.01	−5.57	–	3.21	.60	−3.76	−.05	−8.24	–	0.71	.10
C-A DQ																			
Mothers’ digital media use duration (hours)	None	ref						ref						ref					
	< 1	0.87	.03	−0.28	−	2.03	.14	1.07	.04	−0.44	–	2.59	.16	0.50	.02	−1.22	–	2.22	.57
	1 to < 2	0.72	.02	−0.78	–	2.22	.35	0.35	.01	−1.67	–	2.36	.74	0.73	.02	−1.48	–	2.94	.52
	> 2	−0.30	−.005	−2.48	–	1.88	.79	−0.45	−.01	−3.35	–	2.45	.76	−0.20	−.003	−3.41	–	3.00	.90
L-S DQ																			
Mothers’ digital media use duration (hours)	None	ref						ref						ref					
	< 1	−0.96	−.03	−2.29	–	0.38	.16	−0.61	−.02	−2.40	–	1.18	.50	−1.70	−.06	−3.61	–	0.21	.08
	1 to < 2	−3.07	−.08	−4.81	–	−1.33	< .001	−2.64	−.07	−5.01	–	−0.27	.03	−4.12	−.11	−6.57	–	−1.67	< .001
	> 2	−5.24	−.08	−7.76	–	−2.73	< .001	−5.21	−.08	−8.64	–	−1.79	.003	−5.10	−.08	−8.66	–	−1.55	.01

Partial regression coefficient (B), Standardized partial regression coefficients (β), reference (ref), confidence interval (CI), Kyoto Scale of Psychological Development (KSPD) 2001, Developmental Quotient (DQ), Postural-Motor (P-M), Cognitive-Adaptive (C-A), Language-Social (L-S).

^a^Adjusted for maternal age at delivery; marital status; maternal psychological distress; neuropsychiatric disorders in mothers; maternal bonding failure; maternal education level; paternal education level; maternal occupation; household income; family structure; partner’s frequency of participating in child care activities; frequency of meeting with other parents of same-aged children; number of children including the participant; presence of physical anomalies; attendance at a daycare center or nursery; frequency of reading to the child; number of hours per day the child is allowed to watch TV or DVDs; outdoor playtime during summer and winter.

The results of the analysis stratified by children’s sex revealed that, for both boys and girls, compared with the “none” group, the “one hour or more but less than two hours” (Male B = −2.64; 95% CI −5.01 to −0.27, Female B = −4.12; 95% CI −6.57 to −1.67) and the “two hours or more” (Male B = −5.21; 95% CI −8.64 to −1.79, Female B = −5.10; 95% CI −8.66 to −1.55) groups had lower L-S DQs.

## Discussion

This study investigated the association between the amount of time mothers spent using digital media while with their children and their children’s developmental outcomes at age two (motor, cognitive, language and overall development), as assessed by the KSPD, for a large cohort. In this study, most mothers (78.6%) reported using digital media for less than one hour per day when they were with their children, while 21.4% spent more than one hour on digital media. When mothers used digital media for more than one hour while with their children, the L-S DQs were lower, indicating a dose–response relationship. Additionally, the total DQs were lower when mothers’ digital media use extended to two hours or more.

This study showed that when mothers spent more than two hours a day using digital media while with their children, their total DQ score decreased and was more negatively associated with children’s development. The total DQ is related to the full-scale intelligence quotient of the Wechsler Intelligence Scale for Children-III, which is administered to children aged five and above^38^. Although the difference in total DQ was slight, the long-term effects of mothers’ continuous digital media use on children’s subsequent development remain unclear. This study found no association between maternal digital media use and children’s motor or cognitive development. However, previous studies have shown that there is a positive correlation between maternal digital media use and the duration of children’s digital media use, as well as a negative correlation between children’s digital media use and their cognitive development^39–41^. Furthermore, previous research also revealed a positive correlation between the amount of time children spend using digital media and delays in gross motor development^2^. Although the impact of children’s digital media usage time could not be adjusted for in this study, the impact of TV, which is the digital device most frequently used by this age group, was adjusted for as a covariate^14,15,42^. This study examined the association between mothers’ duration of digital media use while with their children and their children’s motor and cognitive development, while adjusting for children’s screen time as a covariate, and found no association.

Maternal digital media use was also found to be negatively associated with children’s language development. Previous studies reported no association between mothers’ digital media use duration and children’s development^2,14,15^. However, this study yielded different results by focusing on the amount of time mothers spent on digital media while with their children. In this study, digital media use for less than one hour per day was not negatively associated with language development. By contrast, maternal digital media use for more than one hour per day was found to be negatively associated with children’s language development. Key factors in word learning include contiguity (temporal connectedness) and contingency (contextual relevance) with the child’s utterances^43,44^. The qualitative aspects of children’s language experiences, such as turn-taking, may have a greater impact on language development than the quantitative aspects, such as adult word count^43,45^. The problematic use of digital media by parents and “technoference” in parent–child interactions are associated^21^. In a previous Swedish study, 75% of parents of two-year-old children reported using their smartphones at least some of the time during playtime^15^. In a previous study in the United States, 11% of parents reported that on a typical day, they had no technoference in their interactions with their children, while many other parents reported one or more instances of technoference^21^. Parental technoference can adversely affect interactions with children of all ages by diminishing parental attention, responsiveness, and warmth^21,46^. Maternal smartphone use decreases the quality and frequency of interactions with the child and may adversely affect the child’s development of language, cognition, and socioemotional regulation^47^.

While there are negative impacts of parental digital media use on interactions with children, there are also benefits to using digital media. Previous research indicated that parent–child verbal interactions during media use may mitigate the negative impacts on language development, while interactions during educational content may directly enhance language skills^48^. Additionally, digital media offers benefits for the parents of young children, who may use mobile devices to communicate with others, regulate emotions, and alleviate feelings of loneliness^19,49,50^.

We further explored the association between the duration of mothers’ digital media use and the development of their children by sex. No differences were found in the duration of digital media use by mothers between boys and girls. Similarly, previous studies have also found no difference between mothers’ dependence or excessive use of smartphones and the sex of their young children^3,4^. However, we found that girls were more advanced than boys in all developmental outcomes at the age of two, including motor, cognitive, and language. A previous study also reported that girls scored higher on all subjects on the Bayley-III test administered to children aged 18–23 months^51^. However, reports exist that at certain ages, girls scored lower than boys^51^; sex differences in developmental outcomes need to be further examined over time. The sex stratification analysis revealed that mothers’ digital media use exceeding one hour was negatively correlated with children’s language development, while showing no variance between boys and girls. Future studies are needed to determine whether sex differences emerge as young children become older. Notably, when stratified by sex, mothers’ media use beyond two hours showed no association with total DQ. As only 4.9% of mothers in this study reported using digital media for more than two hours daily, the limited sample size may have reduced the power of the stratification analysis.

The quality and quantity of language exposure during childhood, a sensitive period for neuroplasticity, are foundational for subsequent verbal skills, literacy, executive function, mathematical abilities, and social competencies^43^. Cognitive stimulation through reading and shared play at home is deemed critical for fostering interactions^48,52^. In this study, the frequency of reading was controlled as a significant child-rearing environment factor. Nevertheless, maternal digital media use was shown to have a significant negative correlation with child development. Further investigation is required to understand the long-term effects of parental digital media use on children’s development beyond the age of two, including the impact of various child-rearing environments.

This study has several limitations as follows. First, we were unable to determine mothers’ purpose of digital media use and children’s situation during this time (i.e., whether they were using digital media together or were engaged in other activities). Second, we were unable to adjust for the effects of children’s digital media use duration. However, given that television is the most frequently used digital device for this age group^14,15,42^, its effect was adjusted for as a covariate. Third, because mothers’ duration of digital media use was self-reported in the questionnaires, we cannot rule out underreporting^53^. Additional studies employing objective measurement methods are thus required.

The main strength of this study lies in its investigation of the association between mothers’ digital media use duration while with their children and the children’s development, utilizing a large cohort and considering factors such as mother’s mental health, child-rearing environment, and socioeconomic status.

In conclusion, the use of digital media by mothers for more than one hour per day while with their two-year-old children is negatively associated with language development. In addition, use of more than two hours is more negatively associated with their children’s development. We recommend limiting digital media use to less than one hour per day when mothers are with their two-year-old children.

## Data Availability

Data are unsuitable for public deposition due to the ethical restrictions and legal framework of Japan. It is prohibited by the Act on the Protection of Personal Information (Act No. 57 of 30 May 2003, amendment on 9 September 2015) to publicly deposit the data containing personal information. Ethical Guidelines for Medical and Health Research Involving Human Subjects enforced by the Japan Ministry of Education, Culture, Sports, Science and Technology and the Ministry of Health, Labour and Welfare also restrict the open sharing of the epidemiologic data. All inquiries about access to data should be sent to: jecs-en@nies.go.jp. The person responsible for handling enquiries sent to this e-mail address is Dr Shoji F. Nakayama, JECS Programme Office, National Institute for Environmental Studies.
